# Meta-Tree Random Forest: Probabilistic Data-Generative Model and Bayes Optimal Prediction

**DOI:** 10.3390/e23060768

**Published:** 2021-06-18

**Authors:** Nao Dobashi, Shota Saito, Yuta Nakahara, Toshiyasu Matsushima

**Affiliations:** 1 Department of Pure and Applied Mathematics, Waseda University, 3-4-1 Okubo, Shinjuku-ku, Tokyo 169-8555, Japan; nao-0846htt@toki.waseda.jp (N.D.); toshimat@waseda.jp (T.M.); 2 Faculty of Informatics, Gunma University, 4-2, Maebashi, Gunma 371-8510, Japan; 3 Center for Data Science, Waseda University, 1-6-1 Nisniwaseda, Shinjuku-ku, Tokyo 169-8050, Japan; yuta.nakahara@aoni.waseda.jp

**Keywords:** bayes decision theory, data-generative model, meta-tree, prediction, random forest

## Abstract

This paper deals with a prediction problem of a new targeting variable corresponding to a new explanatory variable given a training dataset. To predict the targeting variable, we consider a model tree, which is used to represent a conditional probabilistic structure of a targeting variable given an explanatory variable, and discuss statistical optimality for prediction based on the Bayes decision theory. The optimal prediction based on the Bayes decision theory is given by weighting all the model trees in the model tree candidate set, where the model tree candidate set is a set of model trees in which the true model tree is assumed to be included. Because the number of all the model trees in the model tree candidate set increases exponentially according to the maximum depth of model trees, the computational complexity of weighting them increases exponentially according to the maximum depth of model trees. To solve this issue, we introduce a notion of meta-tree and propose an algorithm called MTRF (Meta-Tree Random Forest) by using multiple meta-trees. Theoretical and experimental analyses of the MTRF show the superiority of the MTRF to previous decision tree-based algorithms.

## 1. Introduction

Various studies in pattern recognition deal with a prediction problem of a targeting variable $y_{n+1}$ corresponding to an explanatory variable $x_{n+1}$ given pairs of explanatory and targeting variable ${\left\{\left(x_{i},y_{i}\right)\right\}}_{i=1}^{n}$. In many of them, the targeting variable $y_{n+1}$ is predicted with a tree *T*. One way to use a tree *T* is to represent a *function* $y_{n+1}=f\left(x_{n+1};T\right)$ and predict $y_{n+1}$. A tree *T* used to represent the function $y_{n+1}=f\left(x_{n+1};T\right)$ is called a decision tree in the literature. In this paper, however, this tree is called a function tree. This is because we distinguish a tree used to represent a function from a tree used to represent a data-generative model (A tree used to represent a data-generative model will be explained in the next paragraph.). In the previous studies, algorithms in which a single function tree is used are discussed in, e.g., CART [1]; algorithms in which multiple function trees are used are discussed, e.g., Random Forest [2] is an algorithm that constructs multiple function trees from ${\left\{\left(x_{i},y_{i}\right)\right\}}_{i=1}^{n}$ and aggregates them to predict $y_{n+1}$ from $x_{n+1}$. There are various extensions of Random Forest, e.g., Generalized Random Forest [3] generalizes the splitting rule of a function tree. Boosting is an algorithm that constructs a function tree sequentially from ${\left\{\left(x_{i},y_{i}\right)\right\}}_{i=1}^{n}$ and combines the constructed function trees to predict $y_{n+1}$ from $x_{n+1}$. There are various Boosting methods, e.g., gradient boosting method in Gradient Boost [4] and XGBoost [5]. Further, previous studies, such as Alternating Decision Forest [6] and Boosted Random Forest [7], combine the ideas of Random Forest and Boosting method. Moreover, combinations of the function trees and neural networks have also been discussed. In Neural Decision Forest [8], inner nodes are replaced by randomized multi-layer perceptrons. In Deep Neural Decision Forest [9], they are replaced by deep convolutional neural networks (CNN). In Adaptive Neural Trees [10], not only their nodes are replaced by CNNs but also their edges are replaced by nonlinear functions. In Deep Forest [11], Random Forests are combined in a cascade structure like deep CNNs. The function tree is also used to understand the deep neural network as an interpretable input-output function in Reference [12]. In these algorithms, a function tree is used to represent the function from $x_{n+1}$ to $y_{n+1}$. Although this function is trained for the data ${\left\{\left(x_{i},y_{i}\right)\right\}}_{i=1}^{n}$ under a certain criterion, statistical optimality for prediction of $y_{n+1}$ is not necessarily discussed theoretically because these algorithms usually do not assume a probabilistic data-generative structure of *x* and *y*. As we will describe later in detail, on the other hand, we assume a probabilistic data-generative structure of *x* and *y*, and consider a statistical optimal prediction of $y_{n+1}$. This is the crucial difference between our study and the related works.

To predict the targeting variable $y_{n+1}$, another way to use a tree *T* is to represent a data-generative model p(y|x,T) that represents a conditional probabilistic structure of *y* given x. A tree *T* used to represent the data-generative model p(y|x,T) is called a model tree throughout this paper. Although not so many studies have assumed the model tree, Reference [13] has assumed it. Because Reference [13] assumed that a targeting variable $y_{n+1}$ is generated according to $p(y|x_{n+1},T)$, statistical optimality for prediction of $y_{n+1}$ can be discussed theoretically. Specifically, based on the Bayes decision theory [14], Reference [13] proposed the optimal prediction of $y_{n+1}$.

In order to explain the optimal prediction based on the Bayes decision theory in more detail, we introduce the terminology *model tree candidate set*. The model tree candidate set is a set of model trees in which the true model tree (When the model tree *T* is used to represent the true data-generative model p(y|x,T), we call it true model tree.) is assumed to be included. One example of a model tree candidate set is a set of model trees whose depth is up to d∈N. The optimal prediction based on the Bayes decision theory is given by weighting all the model trees in the model tree candidate set and the optimal weight of each model tree is given by the posterior probability $p(T|{\left\{\left(x_{i},y_{i}\right)\right\}}_{i=1}^{n})$ of the model tree *T*.

**Figure 1:**
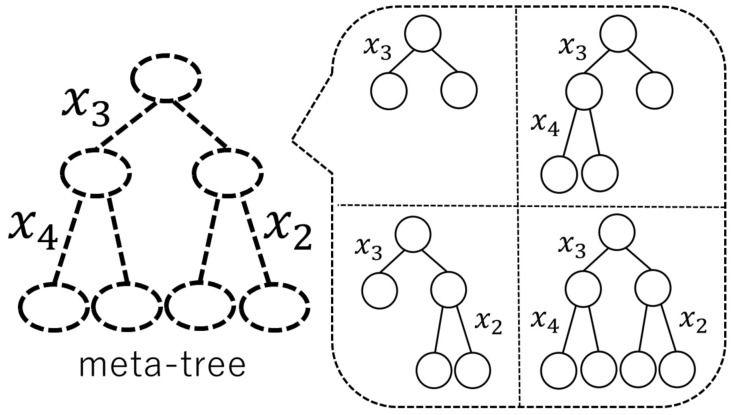
An example of a meta-tree and four model trees represented by it.

Because the number of all the model trees in the model tree candidate set increases *exponentially* according to the maximum depth of model trees, the computational complexity of weighting them increases *exponentially* according to the maximum depth of model trees. One way to reduce the computational complexity is to restrict the model tree candidate set. To represent a restricted model tree candidate set, Reference [13] proposed a notion of *meta-tree*. The concept of a meta-tree was originally used for data compression in information theory (see, e.g., Reference [15]) (Recently, the concept of a meta-tree was also used for image compression [16].). As shown in Figure 1, a model tree candidate set is composed of the model trees represented by the meta-tree.

A meta-tree is also used for the prediction of $y_{n+1}$ in the algorithm proposed by Reference [13]. In summary, a meta-tree has the following two roles: (i) it represents a model tree candidate set; and (ii) it is used for the prediction algorithm. The characteristics of a meta-tree are as follows:

- If the true model tree is in a model tree candidate set represented by a meta-tree, the statistically optimal prediction—optimal prediction based on the Bayes decision theory—can be calculated.
- The number of model trees in a model tree candidate set represented by a meta-tree increases *exponentially* according to the depth of the meta-tree.
- The computational cost in learning processes of a single meta-tree has the same order as that of a single function tree.

Under the assumption that the true model tree is in the restricted model tree candidate set represented by a meta-tree, Reference [13] proposed the optimal prediction based on the Bayes decision theory.

As we have described above, if the true model tree is actually included in the model tree candidate set, the optimal prediction based on the Bayes decision theory is calculated. Hence, it is desirable that we construct the model tree candidate set that includes as many model trees as possible within the allowed computational cost. Motivated by this fact, this paper extends the model tree candidate set compared with Reference [13]. Instead of considering a *single* meta-tree as in Reference [13], we consider *multiple* meta-trees. By using the model tree candidate set represented by multiple meta-trees, we predict $y_{n+1}$ based on the Bayes decision theory. We call this proposed algorithm MTRF(Meta-TreeRandomForest). The characteristics of MTRF are as follows:

- If the true model tree is in a model tree candidate set represented by any of the meta-trees of MTRF, the statistically optimal prediction—optimal prediction based on the Bayes decision theory— can be calculated.
- The number of model trees in the model tree candidate set represented by multiple meta-trees is constant times larger than those contained in a single meta-tree.
- The computational cost in learning processes of meta-trees of MTRF is the same order as that of multiple function trees of Random Forest.

Regarding a meta-tree in Reference [13] or multiple meta-trees of MTRF, how to construct the meta-tree/multiple meta-trees is a problem. One way to solve this issue is to use the function tree-based algorithms; for example, a meta-tree in Reference [13] can be constructed by regarding a function tree in CART as a meta-tree; multiple meta-trees of MTRF can be constructed by regarding function trees in Random Forest as meta-trees. In this way, by regarding a function tree as a meta-tree, our proposed method can be applied to any practical applications in which a function-tree based algorithm is used—insurance claim task (e.g., Reference [5]), letter classification task (e.g., Reference [6]), semantic segmentation (e.g., Reference [8]), face recognition (e.g., Reference [11]), music classification (e.g., Reference [11]), etc.—and we can improve every previous function tree-based algorithm. This is because we can perform a prediction of $y_{n+1}$ by using more model trees compared with the previous algorithms. More detailed discussion will be given in Section 3 and Section 4.

The rest of this paper is organized as follows. In Section 2, we state the preliminaries of our study. Section 3 explains our proposed method—MTRF. In Section 4, we revisit the previous studies, such as CART [1] and Random Forest [2], and compare them with MTRF. Section 5 shows the results of experiments. Section 6 concludes this paper.

## 2. Preliminaries

### 2.1. Model Tree

Let *K* denote the dimension of explanatory variables. The space of feature values is denoted by X:={0,1,…,|X|−1}, and the explanatory variable is denoted by $x:={\left(x_{1},\ldots ,x_{K}\right)}^{⊤}\in X^{K}$. In addition, the space of label values is denoted by Y:={0,1…,|Y|−1}, and the targeting variable is denoted by y∈Y.

In the field of pattern recognition, the probabilistic structure of *x* and *y* is not necessarily assumed. In particular, it is not assumed in most of function tree-based algorithms. In contrast, we consider a triplet of (T,k,θ) defined in Definition 1 below and assume the probabilistic structure p(y|x,θ,T,k) as in Definition 2. We call (T,k) a *model tree* (In Section 1, we called *T* a model tree. More precisely speaking, however, a model tree is (T,k).) and θ a *tree parameter*.

**Definition** **1.** *The notation T denotes the |X|-ary regular (Here, “regular” means that all inner nodes have |X| child nodes.) tree whose depth is up to $D_{\max}$, and T denotes the set of T. Let s be a node of a tree and S be a set of s. The set of the inner nodes of T is denoted by I(T), and the set of the leaf nodes of T is denoted by L(T). Let $k_{s}\in \left\{1,2,\ldots ,K\right\}$ be a component of the feature assign vector of node s. The explanatory variable $x_{k_{s}}\in X$ is assigned to the inner node s∈I(T). Let $k:={\left(k_{s}\right)}_{s\in S}$ denote a feature assign vector, and K denote a set of k. Let $\theta_{s}:=\left(\theta_{0|s},\theta_{1|s},\ldots ,\theta_{|Y|-1|s}\right)\in {\left(0,1\right)}^{\left|Y\right|}$, where $\sum_{y=0}^{|Y|-1}\theta_{y|s}=1$, be a parameter assigned to s∈S. We define $\theta :={\left(\theta_{s}\right)}_{s\in S}$, and* **Θ** *is the set of θ.*

**Definition** **2.** *Because the value of explanatory variable x corresponds to a path from the root node to the leaf node of T whose feature assign vector is k, let s(x)∈L(T) denote the corresponding leaf node. Then, for given x, θ, T, and *k*, the label variable y is generated according to*

(1) $$\begin{array}{c} p\left(y|x,\theta ,T,k\right):=\theta_{y|s(x)}. \end{array}$$

**Figure 2:**
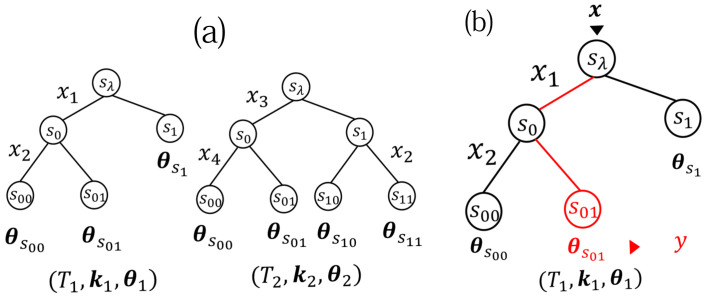
(**a**) Two examples of $\left(T_{1},k_{1},\theta_{1}\right)$ and $\left(T_{2},k_{2},\theta_{2}\right)$, where $T_{1}$ and $T_{2}$ are 2-ary regular trees, $k_{1}=\left(k_{s_{\lambda }},k_{s_{0}}\right)=\left(1,2\right)$, and $k_{2}=\left(k_{s_{\lambda }},k_{s_{0}},k_{s_{1}}\right)=\left(3,4,2\right)$. (**b**) $p\left(y|x={\left(0,1\right)}^{⊤},\theta_{1},T_{1},k_{1}\right)=\theta_{y|s_{01}}$.

Figure 2a shows two examples of (T,k,θ), and Figure 2b illustrates p(y|x,θ,T,k).

### 2.2. Problem Setup

In this subsection, we introduce our problem setup. Let $x_{i}:={\left(x_{i1},\ldots ,x_{iK}\right)}^{⊤}\in X^{K}\left(i=1,\ldots ,n\right)$ denote the value of explanatory variable of the *i*-th data and $x^{n}:=\left(x_{1},\ldots ,x_{n}\right)$. We assume that $x_{1},\ldots ,x_{n}$ are i.i.d. random variables drawn from a probability distribution. Let $y_{i}\in Y\left(i=1,\ldots ,n\right)$ denote the targeting variable of the *i*-th data and $y^{n}:=\left(y_{1},\ldots ,y_{n}\right)$. We assume that $y_{1},\ldots ,y_{n}$ are generated according to p(y|x,θ,T,k) defined in Section 2.1. In regard to this, we assume that the true model tree $\left(T^{★},k^{★}\right)$ and true tree parameter $\theta^{★}$ are unknown, but a model tree candidate set M⊂T×K is known. Note that, as we have explained in Section 1, the model tree candidate set M is a set of model trees (T,k) in which the true model tree is assumed to be included. Further, we assume that a class of parametrized distribution {p(y|x,θ,T,k):θ∈Θ,(T,k)∈M} is known.

The purpose of the prediction problem is to predict unknown targeting variable $y_{n+1}$ corresponding to an explanatory variable $x_{n+1}$ by using the given data $x^{n}$, $y^{n}$, $x_{n+1}$.

### 2.3. Optimal Prediction Based on the Bayes Decision Theory

We introduce a statistically optimal prediction under the problem setup in Section 2.2. From the viewpoint of the Bayes decision theory [14], we shall derive the optimal prediction based on the Bayes decision theory. To this end, we assume priors p(θ), p(T), and p(k).

First, we define a decision function as

(2) $$\begin{array}{c} \delta :X^{Kn}\times Y^{n}\times X^{K}\to Y\;;\left(x^{n},y^{n},x_{n+1}\right)\mapsto \delta \left(x^{n},y^{n},x_{n+1}\right). \end{array}$$

Second, we define a 0–1 loss as follows:

(3) $$\begin{array}{cc} & l\left(y_{n+1},\delta \left(x^{n},y^{n},x_{n+1}\right)\right):=\left\{\begin{array}{c} 0\;(y_{n+1}=\delta \left(x^{n},y^{n},x_{n+1}\right)),\\ 1\;(y_{n+1}\neq \delta \left(x^{n},y^{n},x_{n+1}\right)). \end{array}\right. \end{array}$$

Third, using the 0–1 loss, we define the loss function, which is the expectation of the 0–1 loss taken by new targeting variable $y_{n+1}$:

(4) $$\begin{array}{cc} & L\left(\delta \left(x^{n},y^{n},x_{n+1}\right),\theta ,T,k\right):=\sum_{y_{n+1}\in Y}p\left(y_{n+1}|x_{n+1},\theta ,T,k\right)l\left(y_{n+1},\delta \left(x^{n},y^{n},x_{n+1}\right)\right). \end{array}$$

Next, given the loss function, we define the risk function, which is the expectation of the loss function taken by training data $x^{n}$, $y^{n}$, and $x_{n+1}$:

$$\begin{array}{cc} R & \left(\delta (\cdot ,\cdot ,\cdot ),\theta ,T,k\right) \end{array}$$

(5) $$\begin{array}{c} :=\sum_{x^{n}\in X^{Kn}}\sum_{y^{n}\in Y^{n}}\sum_{x_{n+1}\in X^{K}}p\left(x_{n+1}\right)p\left(x^{n}\right)p\left(y^{n}|x^{n},\theta ,T,k\right)L\left(\delta \left(x^{n},y^{n}x_{n+1}\right),\theta ,T,k\right). \end{array}$$

Finally, the Bayes risk function, which is the expectation of the risk function taken by θ, *T*, and k, is defined as

(6) $$\begin{array}{cc} \mathrm{BR}(\delta (\cdot ,\cdot ,\cdot )):= & \sum_{(T,k)\in M}p\left(k\right)p\left(T\right)\int_{\Theta }p\left(\theta \right)R\left(\delta \left(\cdot ,\cdot ,\cdot \right),\theta ,T,k\right)d\theta . \end{array}$$

Then, we have the following theorem:

**Theorem** **1.** Under the setup in Section 2.2, the decision function $\delta^{*}\left(x^{n},y^{n},x_{n+1}\right)$ that minimizes the Bayes risk function is given as

(7) $$\begin{array}{cc} \delta^{*} & (x^{n},y^{n},x_{n+1})=\\ & \underset{y_{n+1}}{\arg\;\max}\sum_{(T,k)\in M}p\left(k|x^{n},y^{n}\right)p\left(T|x^{n},y^{n},k\right)\int_{\Theta }p\left(\theta |x^{n},y^{n},T,k\right)p\left(y_{n+1}|x_{n+1},\theta ,T,k\right)d\theta . \end{array}$$

The proof of Theorem 1 is in Appendix A. We call $\delta^{*}\left(x^{n},y^{n},x_{n+1}\right)$ the *Bayes decision*.

### 2.4. Previous Study

In this subsection, we introduce how to calculate the Bayes decision in Reference [13]. Because the proof of Reference [13] lacks some explanation, we give more rigorous discussion in comparison. This is one of the contributions in this paper.

**Figure 3:**
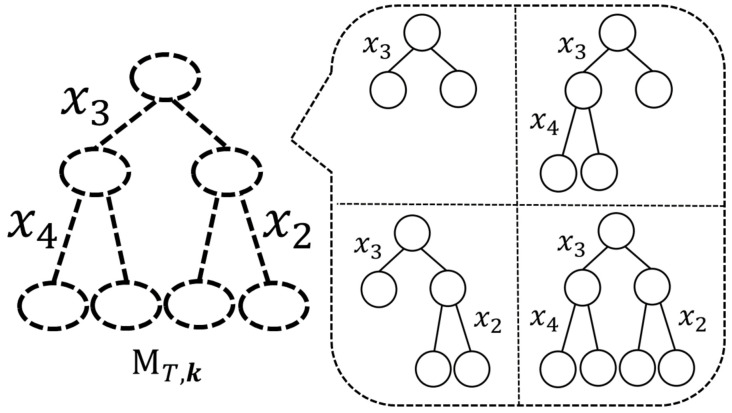
An example of a meta-tree $M_{T,k}$, where *T* is a complete tree with $D_{\max}=2$ and k=(3,4,2).

As we have explained in Section 1, Reference [13] introduced the concept of a “meta-tree” to represent a restricted model tree candidate set. For *T* and k, a meta-tree—denoted by $M_{T,k}$—represents a model tree candidate set that is composed of model trees $\left(T^{\prime },k^{\prime }\right)$ where $T^{\prime }$ is a sub-tree of *T* and $k^{\prime }=k$. An example of $M_{T,k}$ is shown in Figure 3, where *T* is a complete tree with $D_{\max}=2$ and k=(3,4,2). In this example, a meta-tree $M_{T,k}$ represents a model tree candidate set which is composed of four model trees. A meta-tree is also used for the prediction of $y_{n+1}$ in the algorithm proposed by Reference [13]. In short, a meta-tree has the two roles: first, it represents a model tree candidate set, and, second, it is used for the prediction algorithm.

Let $T_{M_{T,k}}$ denote a set of *T* represented by a meta-tree $M_{T,k}$ and let $M=T_{M_{T,k}}\times \left\{k\right\}$. Under the assumption that the true model tree is included in M, the Bayes decision in Theorem 1 is expressed as

(8) $$\begin{array}{cc} \delta^{*} & \left(x^{n},y^{n},x_{n+1}\right)\\ & =\underset{y_{n+1}}{\arg\;\max}\sum_{T^{\prime }\in T_{M_{T,k}}}p\left(T^{\prime }|x^{n},y^{n},k\right)\int_{\Theta }p\left(\theta |x^{n},y^{n},T^{\prime },k\right)p\left(y_{n+1}|x_{n+1},\theta ,T^{\prime },k\right)d\theta . \end{array}$$

Now, (8) can be decomposed into two parts:

(9) $$\begin{array}{cc} 1. & q\left(y_{n+1}|x_{n+1},x^{n},y^{n},T,k\right):=\int_{\Theta }p\left(\theta |x^{n},y^{n},T,k\right)p\left(y_{n+1}|x_{n+1},\theta ,T,k\right)d\theta . \end{array}$$

(10) $$\begin{array}{cc} 2. & \tilde{q}\left(y_{n+1}|x_{n+1},x^{n},y^{n},k\right):=\sum_{T^{\prime }\in T_{M_{T,k}}}p\left(T^{\prime }|x^{n},y^{n},k\right)q\left(y_{n+1}|x_{n+1},x^{n},y^{n},T^{\prime },k\right). \end{array}$$

Reference [13] exactly calculates (9) and (10) under some assumptions. We describe them in Section 2.4.1 and Section 2.4.2, respectively.

#### 2.4.1. Expectation over the Parameters

In this subsection, we explain how to calculate (9). To calculate (9) analytically, a conjugate prior of $\theta_{s}$ given *T* and k, is assumed.

**Assumption** **1.** *We assume $p\left(\theta_{s}|T,k\right)=\mathrm{Dirichlet}\left(\theta_{s}|\alpha_{s}\right)$ for s∈L(T), where $\alpha_{s}$ denotes the hyper-parameter of the Dirichlet distribution.*

Under this assumption, $q(y_{n+1}|x_{n+1},x^{n},y^{n},T,k)$ has a closed form expression and (9) can be calculated exactly. In addition, $q(y_{n+1}|x_{n+1},x^{n},y^{n},T,k)$ has an important property. We describe this in the next lemma as a preparation of Section 2.4.2.

**Lemma** **1.** *For any $T,T^{\prime }\in T$, $x^{n}\in X^{Kn}$, $y^{n}\in Y^{n}$, and $x_{n+1}\in X^{K}$, if $s\left(x_{n+1}\right)\in L\left(T\right)\cap L\left(T^{\prime }\right)$, then*

(11) $$\begin{array}{cc} & q\left(y_{n+1}|x_{n+1},x^{n},y^{n},T,k\right)=q\left(y_{n+1}|x_{n+1},x^{n},y^{n},T^{\prime },k\right). \end{array}$$

The proof of Lemma 1 is in Appendix B. From this lemma, the right- and left-hand sides of (11) are denoted by $q_{s(x_{n+1})}\left(y_{n+1}|x_{n+1},x^{n},y^{n},k\right)$ because they depend on not *T* and $T^{\prime }$ but $s(x_{n+1})$.

#### 2.4.2. Summation over All Model Trees Represented by a Meta-Tree

In this subsection, we explain how to calculate (10). The exact calculation of (10) needs to take summation over all model trees represented by a single meta-tree. However, the computational complexity of this calculation is huge because the number of model trees increases exponentially according to the depth of meta-tree. Reference [13] solved this problem. The advantages of using the method proposed in Reference [13] are as follows:

- This method calculates (10) exactly.
- The computational complexity of calculating (10) in learning parameters is $O(nD_{\max})$, which is the same as that of building a single function tree.

To execute this method, an assumption about the prior probability distribution of *T*—Assumption A2—is required. It should be noted that p(T) is different from the construction method of function trees, given $x^{n}$ and $y^{n}$.

**Assumption** **A2.** *Let $g_{s}\in \left[0,1\right]$ denote a hyper-parameter assigned to any node s∈S. Then, we assume the prior of $T^{\prime }\in T_{M_{T,k}}$ as*

(12) $$\begin{array}{c} p\left(T^{\prime }\right)=\prod_{s\in I(T^{\prime })}g_{s}\prod_{s^{\prime }\in L\left(T^{\prime }\right)}\left(1-g_{s^{\prime }}\right), \end{array}$$

*where $g_{s}=0$ for a leaf node s of a meta-tree $M_{T,k}$.*

**Remark** **1.** *It should be noted that* (12) *is a probability distribution, i.e., $\sum_{T^{\prime }\in T_{M_{T,k}}}p\left(T^{\prime }\right)=1$. See Lemma A1 in Appendix C.*

As we have described in Section 1, a meta-tree is used for the prediction of $y_{n+1}$ in the algorithm proposed by Reference [13]. By using a meta-tree $M_{T,k}$, the recursive function to calculate the Bayes decision (10) is defined as follows.

**Definition** **3.** *We define the following recursive function ${\tilde{q}}_{s}\left(y_{n+1}|x_{n+1},x^{n},y^{n},M_{T,k}\right)$ for any node s on the path in $M_{T,k}$ corresponding to $x_{n+1}$.*

(13) $$\begin{array}{cc} & {\tilde{q}}_{s}\left(y_{n+1}|x_{n+1},x^{n},y^{n},M_{T,k}\right):=\\ & \left\{\begin{array}{cc} q_{s}\left(y_{n+1}|x_{n+1},x^{n},y^{n},k\right), & (\mathrm{if}\;s\;\mathrm{is}\;\mathrm{the}\;\mathrm{leaf}\;\mathrm{node}\;\mathrm{of}\;M_{T,k}),\\ \left(1-g_{s|x^{n},y^{n}}\right)q_{s}\left(y_{n+1}|x_{n+1},x^{n},y^{n},k\right)\\ +g_{s|x^{n},y^{n}}{\tilde{q}}_{s_{\mathrm{child}}}\left(y_{n+1}|x_{n+1},x^{n},y^{n},M_{T,k}\right), & (\mathrm{otherwise}), \end{array}\right. \end{array}$$

*where $s_{\mathrm{child}}$ is the child node of s on the path corresponding to $x_{n+1}$ in $M_{T,k}$, and $g_{s|x^{n},y^{n}}$ is also recursively updated as follows:*

(14) $$\begin{array}{c} g_{s|x^{i},y^{i}}:=\left\{\begin{array}{cc} g_{s}, & (\mathrm{if}\;i=0),\\ \frac{g_{s|x^{i-1},y^{i-1}}{\tilde{q}}_{s_{\mathrm{child}}}\left(y_{i}|x_{i},x^{i-1},y^{i-1},M_{T,k}\right)}{{\tilde{q}}_{s}\left(y_{i}|x_{i},x^{i-1},y^{i-1},M_{T,k}\right)}, & (\mathrm{otherwise}). \end{array}\right. \end{array}$$

Now, (10) can be calculated as shown in the following theorem.

**Theorem** **2.** *$\tilde{q}\left(y_{n+1}|x_{n+1},x^{n},y^{n},k\right)$ can be calculated by*

(15) $$\begin{array}{cc} & \tilde{q}\left(y_{n+1}|x_{n+1},x^{n},y^{n},k\right)={\tilde{q}}_{s_{\lambda }}\left(y_{n+1}|x_{n+1},x^{n},y^{n},M_{T,k}\right), \end{array}$$

*where $s_{\lambda }$ is the root node of $M_{T,k}$. In addition, $p(T|x^{n+1},y^{n+1},k)$ can be calculated as*

(16) $$\begin{array}{c} p\left(T|x^{n+1},y^{n+1},k\right)=\prod_{s\in I(T)}g_{s|x^{n+1},y^{n+1}}\prod_{s^{\prime }\in L\left(T\right)}\left(1-g_{s^{\prime }|x^{n+1},y^{n+1}}\right). \end{array}$$

The proof of Theorem 2 is in Appendix C. Surprisingly, the computational complexity of calculating (10) is $O(nD_{\max})$ by using this theorem.

## 3. Proposed Method

In this section, we introduce our proposed method. Here, we reconsider the general setup where M=T×K. Recall that the Bayes decision (7) is

(17) $$\begin{array}{c} \delta^{*}\left(x^{n},y^{n},x_{n+1}\right)=\underset{y_{n+1}}{\arg\;\max}\sum_{k\in K}p\left(k|x^{n},y^{n}\right)\tilde{q}\left(y_{n+1}|x_{n+1},x^{n},y^{n},k\right), \end{array}$$

where $\tilde{q}\left(y_{n+1}|x_{n+1},x^{n},y^{n},k\right)$ is defined as in (10). In (17), $\tilde{q}\left(y_{n+1}|x_{n+1},x^{n},y^{n},k\right)$ can be calculated in the same way as in Section 2.4. However, regarding the calculation of (17), we need to calculate $\tilde{q}\left(y_{n+1}|x_{n+1},x^{n},y^{n},k\right)$ for all k that satisfies p(k)>0. Because this computational cost increases *exponentially* depending to $D_{\max}$, it is hard to calculate all of them in general. To reduce the computational complexity of (17), we restrict the model tree candidate set to the set represented by multiple meta-trees. The advantages of building multiple meta-trees are as follows:

- As we have explained in Section 2.4.2, the number of model trees represented by each of the meta-trees increases exponentially according to the depth of meta-trees. In addition, the number of model trees in multiple meta-trees is constant times larger than those contained in a single meta-tree.
- If the true model tree is a sub-tree of any of the meta-trees, the statistically optimal prediction—optimal prediction based on the Bayes decision theory—can be calculated.
- If we build *B* meta-trees, the computational cost of it in learning parameters is $O(nBD_{\max})$, which is the same as that of building *B* function trees.

Now, we introduce the next assumption.

**Assumption** **A3.** *For B meta-trees $M_{T_{1},k_{1}},\ldots ,M_{T_{B},k_{B}}$, we define $K^{\prime }\left\{k_{1},\ldots ,k_{B}\right\}$. Then, we assume a uniform distribution on $K^{\prime }$ as a prior probability distribution of k∈K.*

**Figure 4:**
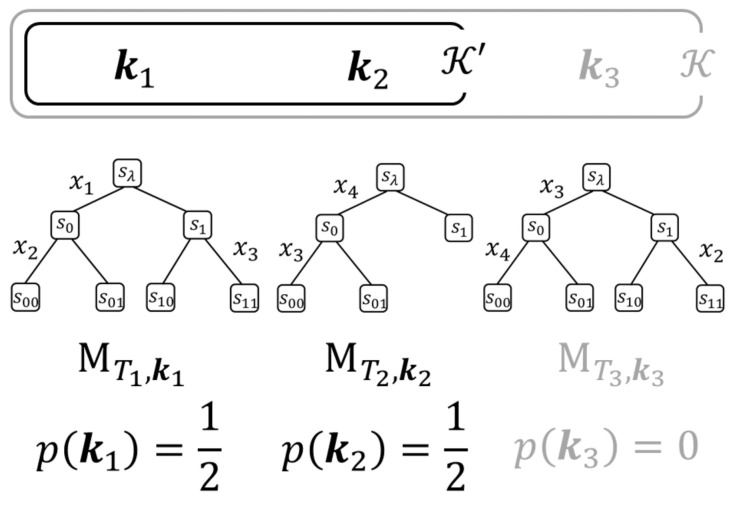
An example of $K=\{k_{1},k_{2},k_{3}\}$ and $K^{\prime }=\left\{k_{1},k_{2}\right\}$. In this example, the prior probability $p\left(k_{1}\right)=p\left(k_{2}\right)=1/2$ and $p(k_{3})=0$.

An example of $K^{\prime }$ is shown in Figure 4.

Next, we prove the following lemma about computing a posterior probability distribution of k. In fact, the recursive function we have defined in Definition 3 is also useful to calculate a posterior probability distribution on $K^{\prime }$ sequentially. The proof of Lemma 2 is in Appendix D.

**Lemma** **2.** *By decomposing $x^{n}$ and $y^{n}$ into the set of i-th data $\left(x_{i},y_{i}\right)\left(i=1,\ldots ,n\right)$ and performing the recursive calculation of ${\tilde{q}}_{s}\left(y_{i}|x_{i},x^{i-1},y^{i-1},M_{T,k}\right)$ as in (13), a posterior probability distribution of $k\in K^{\prime }$ is expressed as follows:*

(18) $$\begin{array}{c} p\left(k|x^{n},y^{n}\right)\propto \prod_{i=1}^{n}{\tilde{q}}_{s_{\lambda }}\left(y_{i}|x_{i},x^{i-1},y^{i-1},M_{T,k}\right). \end{array}$$

From Theorem 2 and Lemma 2, we immediately obtain the next theorem.

**Theorem** **3.** *Let us consider the model tree candidate set represented by B meta-trees, i.e.,*

(19) $$\begin{array}{c} \overset{B}{\underset{b=1}{⋃}}\left(T_{M_{T_{b},k_{b}}}\times \left\{k_{b}\right\}\right). \end{array}$$

*Then, if the true model tree is included in* (19), the Bayes decision $\delta^{*}\left(x^{n},y^{n},x_{n+1}\right)$ can be calculated as

(20) $$\begin{array}{cc} \delta^{*} & \left(x^{n},y^{n},x_{n+1}\right)=\underset{y_{n+1}}{\arg\;\max}\sum_{k\in K^{\prime }}p\left(k|x^{n},y^{n}\right)\tilde{q}\left(y_{n+1}|x_{n+1},x^{n},y^{n},k\right) \end{array}$$

(21) $$\begin{array}{cc} & =\underset{y_{n+1}}{\arg\;\max}\sum_{k\in K^{\prime }}{\tilde{q}}_{s_{\lambda }}\left(y_{n+1}|x_{n+1},x^{n},y^{n},M_{T,k}\right)\prod_{i=1}^{n}{\tilde{q}}_{s_{\lambda }}\left(y_{i}|x_{i},x^{i-1},y^{i-1},M_{T,k}\right). \end{array}$$

*If the true model tree is not included in* (19), *the right-hand side of* (20) *is a sub-optimal prediction of the Bayes decision.*

By using Theorem 3, we can calculate the optimal (possibly sub-optimal) prediction of the Bayes decision effectively. We name this algorithm Meta-Tree Random Forest (MTRF). We summarize MTRF in Algorithm 1.
**Algorithm 1** MTRF**Input:**$x_{n+1},x^{n},y^{n},B,g_{s},\alpha_{s}$**Output:**$\delta^{*}\left(x^{n},y^{n},x_{n+1}\right)$Initialize parameters $g_{s}$.Construct *B* meta-trees $M_{T_{1},k_{1}},\ldots ,M_{T_{B},k_{B}}$ and $K^{\prime }:=\left\{k_{1},\ldots ,k_{B}\right\}$.**for all**$k\in K^{\prime }$**do** **for** i=1,…,n,n+1 **do**  Calculate ${\tilde{q}}_{s_{\lambda }}\left(y_{i}|x_{i},x^{i-1},y^{i-1},M_{T,k}\right)$ with (13).  **if** i≠n+1 **then**   Renew parameters $g_{s|x^{i},y^{i}}$ with (14).  **end if** **end for****end for**Calculate $\delta^{*}\left(x^{n},y^{n},x_{n+1}\right)$ with (20).

**Remark** **2.** *One way to construct multiple meta-trees is to use the ideas of Random Forest. Random Forest builds function trees FT with the impurity (e.g., the entropy and the Gini coefficient) from the training data $x^{n}$ and $y^{n}$. By regarding $\left(T_{1},k_{1}\right)\ldots \left(T_{B},k_{B}\right)$ that are built by Random Forest as meta-trees $M_{T_{1},k_{1}}\ldots M_{T_{B},k_{B}}$, we can construct B meta-trees. In our experiments in Section 5, we adopt this method. The computational complexity of MTRF is $O(nBD_{\max})$ in learning processes, which is the same as that of Random Forest. This is computable unless n, B, or $D_{\max}$ are not extremely huge. In addition, we can parallelize the procedure on each meta-tree.*

## 4. CART and Random Forest Revisited and Comparison with MTRF

### 4.1. CART and Random Forest Revisited

Although CART [1] and Random Forest [2] do not assume the model tree and they use trees to express a function y=f(x;T) (i.e., function tree), they can be regarded as methods of constructing a model tree candidate set.

First, we consider algorithms that use a single function tree, such as CART [1]. Such algorithms can be regarded as selecting a single model tree and predicting $y_{n+1}$ by using the single function tree that corresponds to the single model tree. For example, the prediction of CART—denoted by $\delta_{\mathrm{CART}}\left(x^{n},y^{n},x_{n+1}\right)$—is given by

(22) $$\begin{array}{c} \delta_{\mathrm{CART}}\left(x^{n},y^{n},x_{n+1}\right)=\underset{y_{n+1}}{\arg\;\max}p\left(y_{n+1}|x_{n+1},\hat{\theta }\left(x^{n},y^{n}\right),\hat{T},\hat{k}\right), \end{array}$$

where $\left(\hat{T},\hat{k}\right)$ and $\hat{\theta }\left(x^{n},y^{n}\right)$ denote an estimated model tree and tree parameter, respectively.

Second, as another example, let us consider Random Forest [2]. Random Forest can be regarded as selecting multiple model trees and predicting $y_{n+1}$ by weighting the multiple function trees that correspond to the multiple model trees. It should be noted that the number of function trees is equal to the number of model trees in Random Forest. The prediction of Random Forest—denoted by $\delta_{\mathrm{RF}}\left(x^{n},y^{n},x_{n+1}\right)$—is given by

(23) $$\begin{array}{c} \delta_{\mathrm{RF}}\left(x^{n},y^{n},x_{n+1}\right)=\underset{y_{n+1}}{\arg\;\max}\sum_{\left(T,k\right)\in \hat{M}}\frac{1}{|\hat{M}|}p\left(y_{n+1}|x_{n+1},\hat{\theta }\left(x^{n},y^{n}\right),T,k\right), \end{array}$$

where $\hat{M}=\left\{\left({\hat{T}}_{1},{\hat{k}}_{1}\right),\ldots ,\left({\hat{T}}_{B},{\hat{k}}_{B}\right)\right\}\subset M$ denotes the set of model trees that are constructed by Random Forest.

### 4.2. Comparison of Random Forest with MTRF

Let us consider the situation where the trees that represent the function trees constructed by Random Forest are meta-trees of MTRF. Then, the size of model tree candidate set is far larger in MTRF than in Random Forest.

Random Forest can be regarded as approximating (9), (10) and (17) as in (23). Further, the approximation of the weight $p\left(T,k|x^{n},y^{n}\right)\approx 1/\left|\hat{M}\right|$ is not accurate and the optimality of each function tree’s weight for predicting $y_{n+1}$ has not usually been discussed. In contrast, MTRF calculates the Bayes decision in (9) and (10) exactly and approximates (17) as in (20). Moreover, the optimal weights of *T* and k are given by (16) and (18), respectively.

## 5. Experiments

Most machine learning algorithms require rich numerical experiments to confirm the improvement independent of a specific dataset. However, in our paper, such an improvement is theoretically explained in Section 3 and Section 4. If we redefine all the function trees constructed by any existing method as meta-trees, we can necessarily improve it in a sense of the Bayes risk function since our model tree candidate set contains all the model trees that correspond to the original function trees. This improvement is independent of both datasets and the original methods. Therefore, we perform our experiment only on three types of datasets and compare with the Random Forest. Similar results should be given in any other situation.

### 5.1. Experiment 1

Although the theoretical superiority of MTRF to Random Forest has been explained in Section 3 and Section 4, we performed Experiment 1 to make sure their performance on synthetic data. The procedure of Experiment 1 is the following:

1. We initialize the hyper-parameter $\alpha_{s}=\left(1/|X|,\ldots ,1/|X|\right)$, which is assigned on each node *s*.
2. To make true model trees diverse, we determinate an index of a true feature assign vector k and the true model trees as below:
  - In the true model tree (A), we assign $x_{j}$ to the node whose depth is *j*.
    – true model tree (A-1): the complete 2-ary model tree with depth 4.
    – true model tree (A-2): the regular 2-ary model tree with depth 4. This model tree holds the structure that only left nodes have child nodes and right nodes does not.
  - In the true model tree (B), we assign all different variables to the node.
    – true model tree (B-1): the complete 2-ary model tree with depth 4.
3. We determine parameters $\theta_{s}$ according to the Dirichlet distribution.
4. We generate *K*-dimensional *n* training data from the true model tree and generate *K*-dimensional 100 test data from the true model tree. From (7), Bayes decision takes the same value for any distribution $p(x^{n})$. Therefore, in our experiments, we set a probability distribution of x as follows: Let U({0,1}) denote the uniform distribution on {0,1}. Then, a probability distribution of x is expressed as
  (24) $$\begin{array}{c} p\left(x_{ij}\right)\overset{i.i.d.}{∼}U\left(\left\{0,1\right\}\right)\left(i=1,\ldots ,n,j=1,\ldots ,K\right). \end{array}$$
5. We perform Random Forest that utilizes *B* function trees $\left(T_{1},k_{1}\right),\ldots ,\left(T_{B},k_{B}\right)$. We set its impurity as the entropy and max-depth as $D_{\max}$. Afterward, we make *B* meta-trees $M_{T_{1},k_{1}},\ldots ,M_{T_{B},k_{B}}$. (Of course, their max-depth are $D_{\max}$, too.) Then, we calculate the average prediction error rate of Random Forest and MTRF, where we set the hyper-parameter $g_{s}$ as $g_{s}=1/2$ in MTRF.
6. We repeat Steps 3∼5*Q* times.

**Figure 5:**
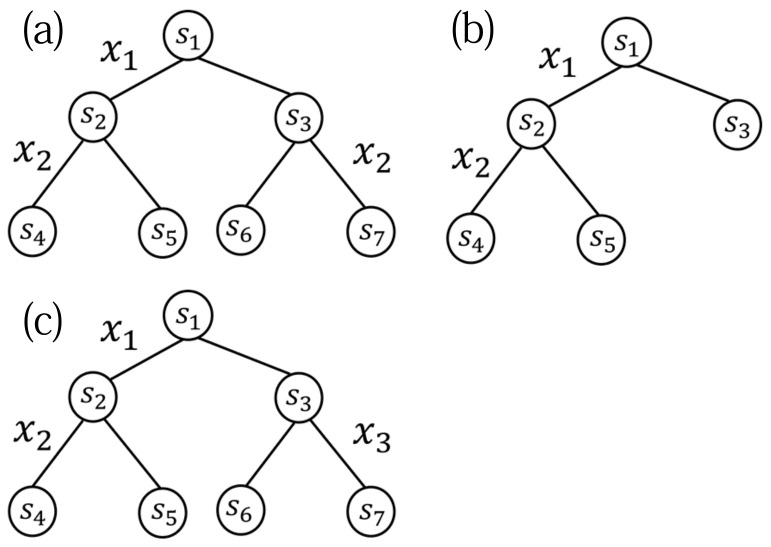
An example of true model tree (A-1) (**a**), model tree (A-2) (**b**), and model tree (B-1) (**c**).

Figure 5 shows an example of these model trees (note that, for simplicity, we illustrate the model trees with depth 2).

**Figure 6:**
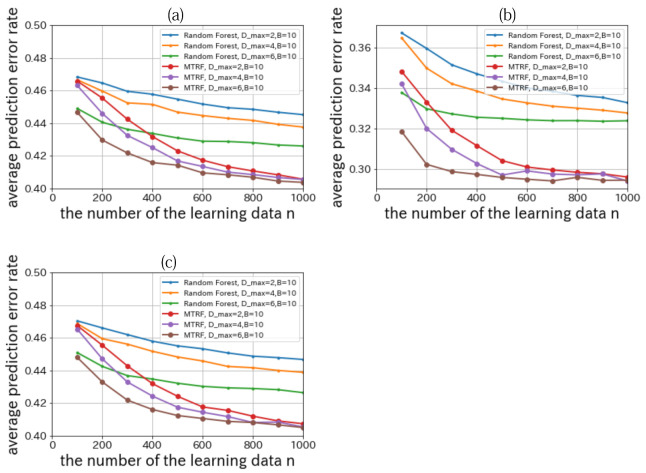
Relationships between the number of learning data *n* and the average prediction error rate where the true model tree is model (A-1) (**a**), model (A-2) (**b**), and model (B-1) (**c**).

We set |X|=|Y|=2, $n=100,200,\ldots ,1000,K=500,B=10,D_{\max}=2,4,6$, and Q=5000. The result is shown in Figure 6.

From Figure 6, when we compare the performance of Random Forest with that of MTRF, we can confirm that MTRF outperforms Random Forest in all conditions. These results are theoretically reasonable because the trees that represent the function trees constructed by Random Forest are meta-trees of MTRF.

### 5.2. Experiment 2

In Experiment 2, we used real data (nursery school data and mushroom data) in the UCI repository (University of California, Irvine Machine Learning Repository). The procedure of Experiment 2 is mostly the same as that of Experiment 1, but the way of sampling data is different; instead of Steps 2, 3, and 4 in Experiment 1, we randomly sampled n+t data from the whole dataset and divided them to *n* training data and *t* test data. We repeated the random sampling of the data for 2500 times.

**Figure 7:**
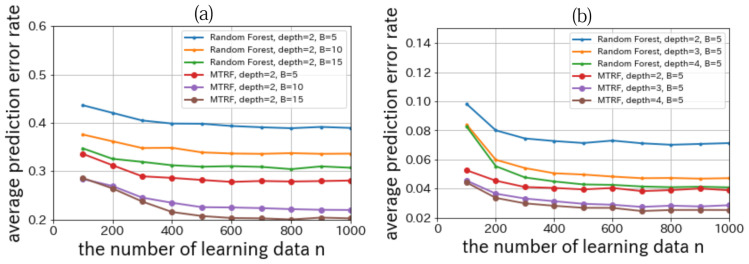
Relationships between the number of the learning data *n* and the average prediction error rate on nursery school data (**a**)/mushroom data (**b**) in UCI repository.

The detail on nursery school data is as follows. The explanatory variables are discrete 8 attributes of a family whose child wants to enter a nursery school (e.g., finance, health, and family structure). The dimension of each attributes is up to 5. The targeting variable is whether the nursery school should welcome him/her or not. We want to consider that a new child should be welcomed with his attributes and learning data. We set |X|=5, |Y|=2, n=100,200,…,1000, t=1000, K=8, B=5, and $D_{\max}=2,3,4$. The result is shown in Figure 7a.

The detail on mushroom data is as follows. The explanatory variables are discrete 22 attributes of a mushroom (e.g., smell, shape, surface, and color). The dimension of each attributes is up to 10. The targeting variable is whether the mushroom can be eaten, not be eaten, or unknown. We want to predict that a new mushroom can be eaten, with its attributes and learning data. We set |X|=22, |Y|=3,n=100,200,…,1000, t=1000, K=22, B=5, and $D_{\max}=2,3,4$. The result is shown in Figure 7b.

From Figure 7, we can confirm MTRF outperforms Random Forest in all conditions.

## 6. Conclusions and Future Work

In this paper, we have considered a model tree and derived the Bayes decision. We have addressed the computational complexity problem of the Bayes decision by introducing a notion of meta-tree, and proposed MTRF (Meta-Tree Random Forest), which uses multiple meta-trees. As we have shown in Theorem 3, if the true model tree is included in the model tree candidate set represented by multiple meta-trees, MTRF calculates the Bayes decision with efficient computational cost. Even if the true model tree is not included in the model tree candidate set represented by multiple meta-trees, MTRF gives the approximation of the Bayes decision. The advantages of MTRF have been theoretically analyzed. In Section 4, we have explained that, if we redefine all the function trees constructed by Random Forest as meta-trees, we can necessarily improve it in a sense of the Bayes risk function since our model tree candidate set contains all the model trees that correspond to the original function trees. We have performed experiments in Section 5 to check the theoretical analysis.

As we have described in Section 1, it is desirable that we construct the model tree candidate set that includes as many model trees as possible within the allowed computational cost. This is because if the true model tree is included in the model tree candidate set, the Bayes decision is calculated. Hence, the main problem of MTRF is how to construct multiple meta-trees. In Section 5, we have constructed multiple meta-trees by using the idea of Random Forest. However, we can consider other ways to construct multiple meta-trees. One of the possible future directions is to use other ideas, such as Boosting. This is one of the future works in our research.

## Figures and Tables

**Figure A1:**
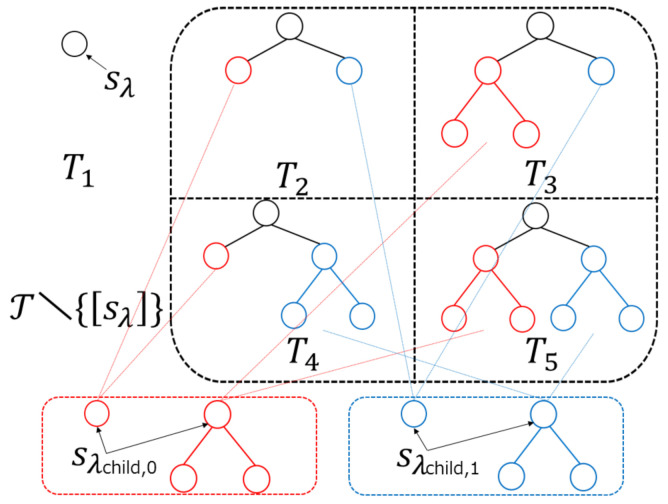
An illustration of $T^{{s_{\lambda }}_{\mathrm{child},0}}$ (left lower enclosure) and $T^{{s_{\lambda }}_{\mathrm{child},1}}$ (right lower enclosure). In the trees $T_{2}$–$T_{5}$, $s_{\lambda }$ has child nodes ${s_{\lambda }}_{\mathrm{child},0}$ and ${s_{\lambda }}_{\mathrm{child},1}$. Therefore, we can decompose the child nodes of $s_{\lambda }$ in the trees $T_{2}$–$T_{5}$ into the nodes included in $T^{{s_{\lambda }}_{\mathrm{child},0}}$ and the nodes included in $T^{{s_{\lambda }}_{\mathrm{child},1}}$.

**Figure A2:**
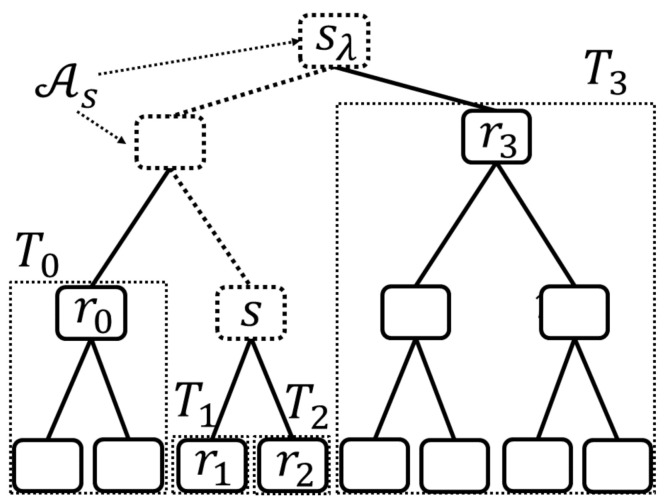
An illustration of $r_{0}$,$r_{1}$,$r_{2}$, and $r_{3}$. In this example, the dotted line represents the path from $s_{\lambda }$ to *s* and $A_{s}$ is the set of two parent nodes of *s*. Furthermore, the rectangles of the dotted line represent four factorized trees $T_{0}$, $T_{1}$, $T_{2}$, and $T_{3}$.

## Data Availability

Publicly available datasets were analyzed in this study. This data can be found here: https://archive.ics.uci.edu/ml/datasets/Nursery and https://archive.ics.uci.edu/ml/datasets/Mushroom (accessed on 8 June 2021).

## Abbreviations

The following abbreviation is used in this manuscript:

MTRF	Meta-Tree Random Forest

## Appendix A. Proof of Theorem 1

**Proof of Theorem 1.** From (4)–(6), and Bayes’ theorem, we have

(A1) $$\begin{array}{cc} \mathrm{BR} & \left(\delta (\cdot ,\cdot ,\cdot )\right)\\ & =\sum_{x^{n}\in X^{Kn}}\sum_{y^{n}\in Y^{n}}\sum_{x_{n+1}\in X^{K}}p\left(x_{n+1}\right)\left(\sum_{y_{n+1}\in Y}p\left(y_{n+1}|x^{n},y^{n},x_{n+1}\right)l\left(y_{n+1},\delta \left(x^{n},y^{n},x_{n+1}\right)\right)\right)\\ & \;\cdot p(x^{n},y^{n}), \end{array}$$

where

(A2) $$\begin{array}{cc} & p\left(y_{n+1}|x^{n},y^{n},x_{n+1}\right)=\sum_{(T,k)\in M}\int_{\Theta }p\left(y_{n+1}|x_{n+1},\theta ,T,k\right)p\left(\theta ,T,k|x^{n},y^{n}\right)d\theta . \end{array}$$

We need to calculate δ that minimizes the brackets in (A1) when we minimize the Bayes risk. Let $a=\delta (x^{n},y^{n},x_{n+1})$ and F(a) denote the formula in the bracket of (A1). Then,

(A3) $$\begin{array}{cc} F(a) & =\sum_{y_{n+1}\in Y}l\left(y_{n+1},a\right)p\left(y_{n+1}|x^{n},y^{n},x_{n+1}\right)\\ & =\sum_{y_{n+1}\in Y\backslash \left\{a\right\}}1\cdot p\left(y_{n+1}|x^{n},y^{n},x_{n+1}\right). \end{array}$$

Therefore, the decision function *a* that minimizes F(a) is

(A4) $$\begin{array}{c} a=\underset{y_{n+1}}{\arg\;\max}p\left(y_{n+1}|x^{n},y^{n},x_{n+1}\right). \end{array}$$

We can prove (7) by substituting (A2) for (A4). □

## Appendix B. Proof of Lemma 1

**Proof of Lemma 1.** Let $\Theta_{s(x_{n+1})}$ denote the space of parameters on $s(x_{n+1})$. In addition, we define

(A5) $$\begin{array}{c} \Theta_{s\neq s(x_{n+1})}\Theta \backslash \Theta_{s(x_{n+1})}. \end{array}$$

Then, we can rewrite the left-hand side of (11) as below:

(A6) $$\begin{array}{cc} q & \left(y_{n+1}|x_{n+1},x^{n},y^{n},T,k\right)=\int_{\Theta }p\left(y_{n+1}|x_{n+1},\theta ,T,k\right)p\left(\theta |x^{n},y^{n},T,k\right)d\theta \\ & =\int_{\Theta_{s(x_{n+1})}}\theta_{y_{n+1}|s\left(x_{n+1}\right)}\int_{\Theta_{s\neq s(x_{n+1})}}p\left(\theta |x^{n},y^{n},T,k\right)d\theta_{s\neq s(x_{n+1})}d\theta_{s(x_{n+1})}, \end{array}$$

where the last equality follows from (1). Looking into the term $\int_{\Theta_{s\neq s(x_{n+1})}}p\left(\theta |x^{n},y^{n},T,k\right)d\theta_{s\neq s(x_{n+1})}$ in (A6), we have

$$\begin{array}{cc} \int_{\Theta_{s\neq s(x_{n+1})}} & p\left(\theta |x^{n},y^{n},T,k\right)d\theta_{s\neq s(x_{n+1})} \end{array}$$

(A7) $$\begin{array}{cc} & \overset{\left(a\right)}{\propto }\int_{\Theta_{s\neq s(x_{n+1})}}p\left(\theta |T,k\right)p\left(y^{n}|x^{n},\theta ,T,k\right)d\theta_{s\neq s(x_{n+1})} \end{array}$$

(A8) $$\begin{array}{cc} & =\int_{\Theta_{s\neq s(x_{n+1})}}\prod_{s\in S}p\left(\theta_{s}|\alpha_{s}\right)\prod_{i=1}^{n}\theta_{y_{i}|s\left(x_{i}\right)}d\theta_{s\neq s(x_{n+1})} \end{array}$$

(A9) $$\begin{array}{cc} & \propto p\left(\theta_{s(x_{n+1})}|\alpha_{s(x_{n+1})}\right)\prod_{i\in \{j\in N:s\left(x_{j}\right)=s\left(x_{n+1}\right)\}}\theta_{y_{i}|s\left(x_{i}\right)}, \end{array}$$

where (a) follows from Bayes’ theorem. Because we can transform the right-hand side of (11) in the same way, we obtain (11). □

## Appendix C. Proof of Theorem 2

**Proof of Theorem 2.** We prove Theorem 2 with mathematical induction according to the following three steps:

Step1: We prove (15) for n=0 with Lemmas A1 and A2.
Step2: We prove (16) for n=0 by using the result of Step 1.
Step3: If we assume (15) and (16) for n=N, we can prove (15) and (16) for n=N+1 by repeating Steps 1 and 2.

In the following, we give a proof of each step.

**Step 1:** First, we prove (15) for n=0. Let $S\left(x_{i}\right)\;\left(i=1,\ldots ,n+1\right)$ denote the set of all nodes on the path of $x_{i}$ in the meta-tree $M_{T,k}$. Let $T_{s}:=\left\{T\in T_{M_{T,k}}|s\in L\left(T\right)\right\}$. Then, we transform $\tilde{q}\left(y_{1}|x_{1},k\right)$ as below:

(A10) $$\begin{array}{cc} \tilde{q}\left(y_{1}|x_{1},k\right) & \overset{\left(a\right)}{=}\sum_{T\in T_{M_{T,k}}}p\left(T\right)q\left(y_{1}|x_{1},T,k\right) \end{array}$$

(A11) $$\begin{array}{cc} & =\sum_{s\in S(x_{1})}\sum_{T\in T_{s}}p\left(T\right)q\left(y_{1}|x_{1},T,k\right) \end{array}$$

(A12) $$\begin{array}{cc} & \overset{\left(b\right)}{=}\sum_{s\in S(x_{1})}q_{s}\left(y_{1}|x_{1},k\right)\sum_{T\in T_{s}}p\left(T\right), \end{array}$$

where (*a*) follows from (10), and (*b*) follows from Lemma 1. The right-hand side of (A12) contains the summation with respect to *T* as below:

(A13) $$\begin{array}{c} \sum_{T\in T_{s}}p\left(T\right). \end{array}$$

Regarding (A13), we prove the following lemmas.

**Lemma** **A1.** *The prior probability distribution*p(T)*assumed in* (12) *satisfies the next property:*

(A14) $$\begin{array}{c} \sum_{T\in T_{M_{T,k}}}p\left(T\right)=1. \end{array}$$

**Proof of Lemma** **A1.** In this proof, for simplicity, we use T instead of $T_{M_{T,k}}$. Before showing the formal proof, we consider an example. Let us consider the set of tree T shown in Figure A1. In this example, T is composed of five model trees. Among these model trees, $T_{1}$ is included in $\left\{\left[s_{\lambda }\right]\right\}$ and $T_{2}$–$T_{5}$ are included in $T\in T\backslash \{\left[s_{\lambda }\right]\}$. Therefore, the next equation holds:

(A15) $$\begin{array}{c} \sum_{T\in T}p\left(T\right)=\sum_{T\in T}\left(\prod_{s\in I(T)}g_{s}\prod_{s^{\prime }\in L\left(T\right)}\left(1-g_{s^{\prime }}\right)\right) \end{array}$$

(A16) $$\begin{array}{c} =\;\left(1\;-\;g_{s_{\lambda }}\right)\;+\;g_{s_{\lambda }}\;\sum_{T\in \{T_{2},T_{3},T_{4},T_{5}\}\}}\;\left(\prod_{s\in L(T)}\left(1\;-\;g_{s}\right)\;\prod_{s^{\prime }\in I\left(T\right)\backslash \left\{s_{\lambda }\right\}}\;g_{s^{\prime }}\;\right)\\ =\;\left(1\;-\;g_{s_{\lambda }}\right)\;+\;g_{s_{\lambda }}\;\left[\left(1-g_{s_{0}}\right)\left(1-g_{s_{1}}\right)+g_{s_{0}}\left(1-g_{s_{00}}\right)\left(1-g_{s_{01}}\right)\left(1-g_{s_{1}}\right)\right. \end{array}$$

(A17) $$\begin{array}{c} \left.+\left(1-g_{s_{0}}\right)g_{s_{1}}\left(1-g_{s_{10}}\right)\left(1-g_{s_{11}}\right)+g_{s_{0}}\left(1-g_{s_{00}}\right)\left(1-g_{s_{01}}\right)g_{s_{1}}\left(1-g_{s_{10}}\right)\left(1-g_{s_{11}}\right)\right] \end{array}$$

(A18) $$\begin{array}{c} =\;\left(1\;-\;g_{s_{\lambda }}\right)\;+\;g_{s_{\lambda }}\;\left[\left\{\left(1-g_{s_{0}}\right)+g_{s_{0}}\left(1-g_{s_{00}}\right)\left(1-g_{s_{01}}\right)\right\}\left\{\left(1-g_{s_{1}}\right)+g_{s_{1}}\left(1-g_{s_{10}}\right)\left(1-g_{s_{11}}\right)\right\}\right] \end{array}$$

(A19) $$\begin{array}{c} \overset{\left(a\right)}{=}\;\left(1\;-\;g_{s_{\lambda }}\right)\;+\;g_{s_{\lambda }}\;\left[\left\{\left(1-g_{s_{0}}\right)+g_{s_{0}}\right\}\left\{\left(1-g_{s_{1}}\right)+g_{s_{1}}\right\}\right] \end{array}$$

(A20) $$\begin{array}{c} =1, \end{array}$$

where (a) follows from $g_{s}=0$ for a leaf node *s* of the meta-tree (see Assumption A2). Now, we give the formal proof. Because

(A21) $$\begin{array}{c} \sum_{T\in T}p\left(T\right)=\sum_{T\in T}\left(\prod_{s\in I(T)}g_{s}\prod_{s^{\prime }\in L\left(T\right)}\left(1-g_{s^{\prime }}\right)\right), \end{array}$$

we prove

(A22) $$\begin{array}{c} \sum_{T\in T}\left(\prod_{s\in I(T)}g_{s}\prod_{s^{\prime }\in L\left(T\right)}\left(1-g_{s^{\prime }}\right)\right)=1 \end{array}$$

by induction. Let $\left[s_{\lambda }\right]$ denote the tree that consists of only the root node $s_{\lambda }$ of *T*. Then, we have

(A23) $$\begin{array}{cc} \sum_{T\in T} & \;\left(\prod_{s\in L(T)}\left(1\;-\;g_{s}\right)\;\prod_{s^{\prime }\in I\left(T\right)}g_{s^{\prime }}\;\right)\\ & =\;\left(1\;-\;g_{s_{\lambda }}\right)\;+\;g_{s_{\lambda }}\;\sum_{T\in T\backslash \{\left[s_{\lambda }\right]\}}\;\left(\prod_{s\in L(T)}\left(1\;-\;g_{s}\right)\;\prod_{s^{\prime }\in I\left(T\right)\backslash \left\{s_{\lambda }\right\}}\;g_{s^{\prime }}\;\right). \end{array}$$

Because each tree $T\in T\backslash \{\left[s_{\lambda }\right]\}$ is identified by |X| sub-trees whose root nodes are the child nodes of $s_{\lambda }$, let ${s_{\lambda }}_{\mathrm{child},j}$ denote the *j*-th child node of $s_{\lambda }$ for 0≤j≤|X|−1 and $T^{{s_{\lambda }}_{\mathrm{child},j}}$ denote the set of sub-trees whose root node is ${s_{\lambda }}_{\mathrm{child},j}$. Figure A1 shows an example of $T^{{s_{\lambda }}_{\mathrm{child},0}}$ and $T^{{s_{\lambda }}_{\mathrm{child},1}}$. Then, the summation in the right-hand side of (A23) is factorized as

(A24) $$\begin{array}{c} \prod_{j=0}^{|X|-1}\sum_{T_{j}\in T^{{s_{\lambda }}_{\mathrm{child},j}}}\;\left(\prod_{s\in L(T_{j})}\;\left(1\;-\;g_{s}\right)\;\prod_{s^{\prime }\in I\left(T_{j}\right)}\;g_{s^{\prime }}\;\right). \end{array}$$

Consequently, because the goal is to show (A22), Lemma A1 is proven by induction. □

**Lemma** **A2.**
*Let $A_{s}$ denote the set of all ancestor nodes of s. Then, we have*

(A25) $$\begin{array}{cc} & \sum_{T\in T_{s}}p\left(T\right)=\left(1-g_{s}\right)\prod_{s^{\prime }\in A_{s}}g_{s^{\prime }}. \end{array}$$

**Proof of Lemma** **A2.**

(A26) $$\begin{array}{cc} \sum_{T\in T_{s}}p\left(T\right) & \overset{\left(a\right)}{=}\sum_{T\in T_{s}}\prod_{s^{\prime }\in I\left(T\right)}g_{s^{\prime }}\prod_{s^{\prime \prime }\in L\left(T\right)}\left(1-g_{s^{\prime \prime }}\right) \end{array}$$

(A27) $$\begin{array}{cc} & \overset{\left(b\right)}{=}\left(1-g_{s}\right)\prod_{s^{\prime }\in A_{s}}g_{s^{\prime }}\sum_{T\in T_{s}}\prod_{s^{\prime \prime }\in I\left(T\right)\backslash A_{s}}g_{s^{\prime \prime }}\prod_{s^{\prime \prime \prime }\in L\left(T\right)\backslash \left\{s\right\}}\left(1-g_{s^{\prime \prime \prime }}\right), \end{array}$$

where (*a*) follows from Assumption 2, and (*b*) follows from the fact that all trees $T\in T_{s}$ have the node *s* and its ancestor nodes. Further, as we will prove later, it holds that

(A28) $$\begin{array}{c} \sum_{T\in T_{s}}\prod_{s^{\prime \prime }\in I\left(T\right)\backslash A_{s}}g_{s^{\prime \prime }}\prod_{s^{\prime \prime \prime }\in L\left(T\right)\backslash \left\{s\right\}}\left(1-g_{s^{\prime \prime \prime }}\right)=1. \end{array}$$

Thus, we obtain (A25) by combining (A26) and (A28). To prove (A28), we can apply the factorization of Lemma A1 to (A28) if we regard the node whose parent node is *s* or an element of $A_{s}$ as a root node, as shown in Figure A2. Let *J* denote the number of that factorized trees, and let $r_{j}$ denote the root node of *j*-th factorized tree. Then, we can rewrite (A28) as follows:

(A29) $$\begin{array}{cc} & \sum_{T\in T_{s}}\prod_{s^{\prime \prime }\in I\left(T\right)\backslash A_{s}}g_{s^{\prime \prime }}\prod_{s^{\prime \prime \prime }\in L\left(T\right)\backslash \left\{s\right\}}\left(1-g_{s^{\prime \prime \prime }}\right)=\prod_{j=0}^{J-1}\sum_{T_{j}\in T^{r_{j}}}\;\left(\prod_{s\in L(T_{j})}\;\left(1\;-\;g_{s}\right)\;\prod_{s^{\prime }\in I\left(T_{j}\right)}\;g_{s^{\prime }}\;\right)=1. \end{array}$$

Hence, the proof is complete. □

Now, we transform (A12) as follows.

(A30) $$\begin{array}{cc} \sum_{s\in S(x_{1})}q_{s}\left(y_{1}|x_{1},k\right)\sum_{T\in T_{s}}p\left(T\right) & \overset{\left(a\right)}{=}\sum_{s\in S(x_{1})}q_{s}\left(y_{1}|x_{1},k\right)\left(1-g_{s}\right)\prod_{s^{\prime }\in A_{s}}g_{s^{\prime }}\\ & =\left(1-g_{s_{\lambda }}\right)q_{s_{\lambda }}\left(y_{1}|x_{1},k\right) \end{array}$$

(A31) $$\begin{array}{cc} & +g_{s_{\lambda }}\sum_{s\in S\left(x_{1}\right)\backslash \left\{s_{\lambda }\right\}}q_{s}\left(y_{1}|x_{1},k\right)\left(1-g_{s}\right)\prod_{s^{\prime }\in A_{s}\backslash \left\{s_{\lambda }\right\}}g_{s^{\prime }}, \end{array}$$

where (*a*) follows from Lemma A2.

Here, let $s_{\lambda_{\mathrm{child}}}$ denote the node that is a child node of $s_{\lambda }$ and an element of $S(x_{1})$. Then, we can decompose

(A32) $$\begin{array}{cc} & \sum_{s\in S\left(x_{1}\right)\backslash \left\{s_{\lambda }\right\}}q_{s}\left(y_{1}|x_{1},k\right)\left(1-g_{s}\right)\prod_{s^{\prime }\in A_{s}\backslash \left\{s_{\lambda }\right\}}g_{s^{\prime }}\\ & =\left(1-g_{{s_{\lambda }}_{\mathrm{child}}}\right)q_{{s_{\lambda }}_{\mathrm{child}}}\left(y_{1}|x_{1},k\right)\\ & +g_{{s_{\lambda }}_{\mathrm{child}}}\sum_{s\in S\left(x_{1}\right)\backslash \left\{s_{\lambda },{s_{\lambda }}_{\mathrm{child}}\right\}}q_{s}\left(y_{1}|x_{1},k\right)\left(1-g_{s}\right)\prod_{s^{\prime }\in A_{s}\backslash \left\{s_{\lambda },{s_{\lambda }}_{\mathrm{child}}\right\}}g_{s^{\prime }}. \end{array}$$

The equation (A32) has a similar structure to (A31). Like this, we can decompose (A31) recursively to the leaf node that is included in $S(x_{1})$. In addition, we can utilize the assumption $g_{s}=0$ for any leaf nodes *s* of a meta-tree $M_{T,k}$. Consequently, we can prove that (A31) coincides with ${\tilde{q}}_{s_{\lambda }}\left(y_{1}|x_{1},M_{T,k}\right)$.

**Step 2:** Next, we prove (16) for n=0. Because the parameters $g_{s}$ are updated only when their nodes are the elements of $S(x_{1})$, we decompose the right-hand side of (16) for n=0 as follows:

$$\begin{array}{cc} & \prod_{s\in I(T)}g_{s|x^{1},y^{1}}\prod_{s^{\prime }\in L\left(T\right)}\left(1-g_{s^{\prime }|x^{1},y^{1}}\right)\\ & =\prod_{s\in I\left(T\right)\cap S\left(x_{1}\right)}g_{s|x^{1},y^{1}}\prod_{s^{\prime }\in L\left(T\right)\cap S\left(x_{1}\right)}\left(1-g_{s^{\prime }|x^{1},y^{1}}\right) \end{array}$$

(A33) $$\begin{array}{cc} & \cdot \prod_{s^{\prime \prime }\in I\left(T\right)\backslash S\left(x_{1}\right)}g_{s^{\prime \prime }|x^{1},y^{1}}\prod_{s^{\prime \prime \prime }\in L\left(T\right)\backslash S\left(x_{1}\right)}\left(1-g_{s^{\prime \prime \prime }|x^{1},y^{1}}\right) \end{array}$$

(A34) $$\begin{array}{cc} & =\prod_{s\in I\left(T\right)\cap S\left(x_{1}\right)}g_{s|x^{1},y^{1}}\prod_{s^{\prime }\in L\left(T\right)\cap S\left(x_{1}\right)}\left(1-g_{s^{\prime }|x^{1},y^{1}}\right)\prod_{s^{\prime \prime }\in I\left(T\right)\backslash S\left(x_{1}\right)}g_{s^{\prime \prime }}\prod_{s^{\prime \prime \prime }\in L\left(T\right)\backslash S\left(x_{1}\right)}\left(1-g_{s^{\prime \prime \prime }}\right). \end{array}$$

We transform the part of (A34), whose parameters $g_{s}$ are renewed by $x^{1}$ and $y^{1}$, as follows:

$$\begin{array}{cc} \prod_{s\in I\left(T\right)\cap S\left(x_{1}\right)}g_{s|x^{1},y^{1}} & \prod_{s^{\prime }\in L\left(T\right)\cap S\left(x_{1}\right)}\left(1-g_{s^{\prime }|x^{1},y^{1}}\right) \end{array}$$

(A35) $$\begin{array}{cc} & \overset{\left(a\right)}{=}\left(1-g_{s\left(x_{1}\right)|x^{1},y^{1}}\right)\prod_{s\in I\left(T\right)\cap S\left(x_{1}\right)}g_{s|x^{1},y^{1}} \end{array}$$

(A36) $$\begin{array}{cc} & \overset{\left(b\right)}{=}\left(1-g_{s(x_{1})}\right)\prod_{s\in I\left(T\right)\cap S\left(x_{1}\right)}\frac{g_{s}{\tilde{q}}_{s_{\mathrm{child}}}\left(y_{1}|x_{1},M_{T,k}\right)}{{\tilde{q}}_{s}\left(y_{1}|x_{1},M_{T,k}\right)} \end{array}$$

(A37) $$\begin{array}{cc} & \overset{\left(c\right)}{=}\frac{\left(1-g_{s(x_{1})}\right){\tilde{q}}_{s(x_{1})}\left(y_{1}|x_{1},M_{T,k}\right)}{{\tilde{q}}_{s_{\lambda }}\left(y_{1}|x_{1},M_{T,k}\right)}\prod_{s\in I\left(T\right)\cap S\left(x_{1}\right)}g_{s} \end{array}$$

(A38) $$\begin{array}{cc} & =\frac{{\tilde{q}}_{s(x_{1})}\left(y_{1}|x_{1},M_{T,k}\right)}{{\tilde{q}}_{s_{\lambda }}\left(y_{1}|x_{1},M_{T,k}\right)}\prod_{s\in I\left(T\right)\cap S\left(x_{1}\right)}g_{s}\prod_{s^{\prime }\in L\left(T\right)\cap S\left(x_{1}\right)}\left(1-g_{s^{\prime }}\right), \end{array}$$

where (*a*) follows from $s^{\prime }\in L\left(T\right)\cap S\left(x_{1}\right)$ is uniquely determined as $s(x_{1})$, (*b*) follows from Definition 3, and (*c*) follows from reduction of ${\tilde{q}}_{s}\left(y_{1}|x_{1},M_{T,k}\right)$.

Thus, the right-hand side of (A34) is calculated as

(A39) $$\begin{array}{cc} & \frac{{\tilde{q}}_{s(x_{1})}\left(y_{1}|x_{1},M_{T,k}\right)}{{\tilde{q}}_{s_{\lambda }}\left(y_{1}|x_{1},M_{T,k}\right)}\prod_{s\in I(T)}g_{s}\prod_{s^{\prime }\in L\left(T\right)}\left(1-g_{s^{\prime }}\right). \end{array}$$

(A40) $$\begin{array}{cc} & \overset{\left(a\right)}{=}\frac{{\tilde{q}}_{s(x_{1})}\left(y_{1}|x_{1},M_{T,k}\right)p\left(T\right)}{{\tilde{q}}_{s_{\lambda }}\left(y_{1}|x_{1},M_{T,k}\right)} \end{array}$$

(A41) $$\begin{array}{cc} & \overset{\left(b\right)}{=}\frac{q\left(y_{1}|x_{1},T,k\right)p\left(T\right)}{\tilde{q}\left(y_{1}|x_{1},k\right)} \end{array}$$

(A42) $$\begin{array}{cc} & \overset{\left(c\right)}{=}p\left(T|x^{1},y^{1},k\right), \end{array}$$

where (a) follows from Assumption 2, the denominator of (b) follows from Theorem 2 for n=0, the numerator of (b) follows from Definition 3 and the definition of $q_{s(x_{n+1})}\left(y_{n+1}|x_{n+1},x^{n},y^{n},k\right)$, and (c) follows from the Bayes’ theorem.

**Step 3:** Finally, if we assume (15) and (16) are true when n=N, we can also prove (15) and (16) when n=N+1 in the same way. Therefore, Theorem 2 holds.

## Appendix D. Proof of Lemma 2

**Proof of Lemma** **2.**

(A43) $$\begin{array}{cc} p(k|x^{n},y^{n}) & \propto p\left(k\right)p\left(x^{n},y^{n}|k\right) \end{array}$$

(A44) $$\begin{array}{cc} & =p\left(k\right)\prod_{i=1}^{n}\tilde{q}\left(y_{i}|x_{i},x^{i-1},y^{i-1},k\right)p\left(x_{i}|x^{i-1},y^{i-1},k\right) \end{array}$$

(A45) $$\begin{array}{cc} & \overset{\left(a\right)}{\propto }\prod_{i=1}^{n}\tilde{q}\left(y_{i}|x_{i},x^{i-1},y^{i-1},k\right) \end{array}$$

(A46) $$\begin{array}{cc} & \overset{\left(b\right)}{=}\prod_{i=1}^{n}{\tilde{q}}_{s_{\lambda }}\left(y_{i}|x_{i},x^{i-1},y^{i-1},M_{T,k}\right), \end{array}$$

where (a) follows from Assumption A3 and $x_{1},\ldots ,x_{n}$ are i.i.d.; (b) follows from Theorem 2 at each i=1,…,n. □
